# Supplementary material for: Asbestos Exposure and Malignant Mesothelioma in Construction Workers—Epidemiological Remarks by the Italian National Mesothelioma Registry (ReNaM)
Source: Int J Environ Res Public Health. 2021 Dec 26;19(1):235. doi: 10.3390/ijerph19010235 (PMC8744912; doi:10.3390/ijerph19010235)
Supplement: Supplementary file 1 [file ijerph-19-00235-s001.zip › ijerph-1513215-supplementary.pdf]

Asbestos Exposure and Malignant Mesothelioma in Construction Workers—Epidemiological Remarks by the Italian National Mesothelioma Registry (ReNaM)

Members of the ReNaM working group: Massari S [1], Iavicoli S [1], Signorini S [1] (INAIL); Detragiache E [2]; Richiardi L [3], Mirabelli D [3], Gangemi M [3], Stura A [3], Brentisci C [3], Gilardetti M [3]; Campi MG [4], Mazzucco G [4], Canessa PA [4]; Dallari B [5], Pesatori AC [5], Riboldi L [5], Rugarli S [5], Zellino C [5]; Fedeli U [8], Merler E [8], Girardi P [8], Casotto V [8] (COR Veneto); D'Agostin F [9], De Michieli P [9]; Mangone L [10], Storchi C [10], Sala O [10]; Badiali AM [11], Baldassarre A [11], Cacciarini V [11], Giovannetti L [11], Martini A [11]; Pascucci C [12], Calisti R [12]; Stracci F [13], Bodo P [13], Latini L [13], Sarnari L [13]; Carai A [14], Ascoli V [14], Michelozzi P [14], Davoli M [14], Forastiere F [14], Cavariani F [14], Ancona L [14]; Serio G [18], De Maria L [18], Caputi A [18], Delfino MC [18], Pentimone F [18], Luisi V [18]; Lio SG [20]; Cascone G [21], Frasca G [21], Giurdanella MC [21], Martorana C [21], Rollo P [21], Spata E [21], Dardanoni G [21], Scondotto S [21]; Angius M [22] and Stecchi S [22]

[1] Department of Occupational and Environmental Medicine, Epidemiology and Hygiene, Istituto Nazionale per l'Assicurazione Contro gli Infortuni sul Lavoro, 00143 Roma, Italy

[2] Valle d'Aosta Health Local Unit, Regional Operating Center of Valle d'Aosta (COR Valle d'Aosta), 11100 Aosta, Italy

[3] Unit of Cancer Epidemiology, Regional Operating Center of Piemonte (COR Piemonte), University of Torino and CPO-Piemonte, 10124 Torino, Italy

[4] Regional Operating Center of Liguria (COR Liguria), UO Clinical Epidemiology, IRCCS AOU Policlinico San Martino, 16132 Genova, Italy; lucia.benfatto@hsanmartino.it (L.B.); davide.malacarne@hsanmartino.it (D.M.)

[5] Epidemiology Unit, Regional Operating Center of Lombardia (COR Lombardia), Fondazione IRCCS Ca' Granda Ospedale Maggiore Policlinico, 20122 Milano, Italy

[6] Hygiene and occupational medicine, Provincial Unit of Health, Regional Operating Center of Autonomous Province of Trento (COR A.P. of Trento), 38100 Trento, Italy

[7] Occupational Medicine Unit, Alto Adige Health Authority, Regional Operating Center of Autonomous Province of Bolzano (COR A.P. of Bolzano), 39100 Bolzano, Italy

[8] Azienda Zero, Epidemiological Department, Regional Operating Center of Veneto (COR Veneto), Veneto Region, 35131 Padova, Italy

[9] Clinical Unit of Occupational Medicine, Regional Operating Center of Friuli-Venezia Giulia (COR Friuli-Venezia Giulia), University of Trieste -Trieste General Hospitals, 34123 Trieste, Italy

[10] Health Local Unit, Public Health Department, Regional Operating Center of Emilia-Romagna (COR Emilia-Romagna), 42020 Reggio Emilia, Italy;

[11] Prevention and Clinical Network, Institute for Cancer Research, Regional Operating Center of Toscana (COR Toscana), 50139 Firenze, Italy

[12] Regional Operating Center of Marche (COR Marche), School of Medicinal and Health Products Sciences, University of Camerino, 62032 Camerino, Italy

[13] Regional Operating Center of Umbria (COR Umbria), Servizio Prevenzione, Sanità Veterinaria e Sicurezza Alimentare–Regione Umbria, 06126 Perugia, Italy

[14] Regional Operating Center of Lazio (COR Lazio), Lazio Region, Department of Epidemiology, 00143 Roma, Italy

[15] Occupational Medicine Unit, Health Local Unit, Regional Operating Center of Abruzzo (COR Abruzzo), 65121 Pescara, Italy

[16] Oncology Unit, Cardarelli Hospital, Regional Operating Center of Molise (COR Molise), Campobasso 86100, Italy

[17] Department of Experimental Medicine, "Luigi Vanvitelli" University, Regional Operating Center of Campania (COR Campania), 80138 Napoli, Italy

[18] Section of Occupational Medicine ''B.Ramazzini'', Department of Interdisciplinary Medicine, Regional Operating Center of Puglia (COR Puglia), University of Bari, 70125 Bari, Italy

[19] Epidemiologic Regional Center, Regional Operating Center of Basilicata (COR Basilicata), 85100 Potenza, Italy
[20] Public Health Unit, Regional Operating Center of Calabria (COR Calabria), 88900 Crotone, Italy
[21] Cancer Registry ASP Ragusa and Sicilia Regional Epidemiological Observatory, Regional Operating Center of Sicilia (COR Sicilia), 97100 Ragusa, Italy
[22] Regional Epidemiological Center, Regional Operating Center of Sardegna (COR Sardegna), 09125 Cagliari, Italy
